# Determination of Dielectric Properties of Cells using AC Electrokinetic-based Microfluidic Platform: A Review of Recent Advances

**DOI:** 10.3390/mi11050513

**Published:** 2020-05-19

**Authors:** Wenfeng Liang, Xieliu Yang, Junhai Wang, Yuechao Wang, Wenguang Yang, Lianqing Liu

**Affiliations:** 1 School of Mechanical Engineering, Shenyang Jianzhu University, Shenyang 110168, China; yang.xieliu@sjzu.edu.cn (X.Y.); jhwang@sjzu.edu.cn (J.W.); 2 State Key Laboratory of Robotics, Shenyang Institute of Automation, Chinese Academy of Sciences, Shenyang 110016, China; ycwang@sia.cn; 3 School of Electromechanical and Automotive Engineering, Yantai University, Yantai 264005, China; yangwenguang@ytu.edu.cn

**Keywords:** electro-rotation, dielectrophoresis, optically-induced dielectrophoreis, alternating current (AC) electrokinetic, cell dielectric properties, microfluidic

## Abstract

Cell dielectric properties, a type of intrinsic property of cells, can be used as electrophysiological biomarkers that offer a label-free way to characterize cell phenotypes and states, purify clinical samples, and identify target cancer cells. Here, we present a review of the determination of cell dielectric properties using alternating current (AC) electrokinetic-based microfluidic mechanisms, including electro-rotation (ROT) and dielectrophoresis (DEP). The review covers theoretically how ROT and DEP work to extract cell dielectric properties. We also dive into the details of differently structured ROT chips, followed by a discussion on the determination of cell dielectric properties and the use of these properties in bio-related applications. Additionally, the review offers a look at the future challenges facing the AC electrokinetic-based microfluidic platform in terms of acquiring cell dielectric parameters. Our conclusion is that this platform will bring biomedical and bioengineering sciences to the next level and ultimately achieve the shift from lab-oriented research to real-world applications.

## 1. Introduction 

The intrinsic properties of cells, such as the geometrical parameters [1,2], refractive index [3,4], stiffness [5], Young modulus [6], and dielectric parameters [7], have received widespread attention in bio-related fields. More recently, the degradation of cellular mechanics and morphology has been investigated to predict the biological age of cells and ultimately reveal the ageing process and chronic disease states of older adults [8]. Furthermore, researchers have discovered that metastatic cancer cells are more than 70% softer than normal cells, meaning that cellular stiffness can be used for the label-free, early, and real-time detection of cancer cells, although normal cells and cancer cells may have similar sizes [9]. Using the differences in dielectric properties between different cells, the separation of Raji cells from red blood cells (RBCs) can be achieved without the aid of any biochemical labels [10], which offers an alternative solution to characterizing clinical cancer treatments by anti-cancer drugs. In addition, based on the density and compressibility differences between cells, exosomes can be isolated from whole blood with a purity of over 99.999%, which can also be accomplished in a label-free and contact-free manner [11]. The real-time characterization of the deformation parameter of RBCs reveals that the increase in the cytoskeletal tension of RBCs and the decrease in the bending modulus of RBCs are directly correlated with the parasite invasion efficiency, thereby implying the occurrence of malaria parasite invasion [12]. It has been demonstrated that cell membrane capacitance, a type of cell dielectric parameter, can be used for the real-time, label-free, and contact-free monitoring of stem cell differentiation [13]. Additionally, whole cell membrane capacitance serving as a label-free biomarker can be employed to monitor stem cell fate and differentiation, thereby opening new avenues for regulating stem cell fate and continued progress as well as enhancing our understanding of basic stem cell biology [14]. Most importantly, the label-free and non-invasive isolation and enrichment of the circulating tumor cells (CTCs) derived from clinical samples is another application of the extracted cell dielectric properties, which will give valuable prognostic guides for treating malignant diseases and detecting the metastasis and deterioration of tumors [15]. Hence, cell dielectric properties, a type of intrinsic property of cells, can be used as electrophysiological biomarkers that offer a label-free way to characterize cell phenotypes and states, purify clinical samples, and identify target cancer cells. To sum up, research into the intrinsic properties of cells has enabled significant progress in the biomedical, bioengineering, and drug research fields.

The above-mentioned results are supported by a variety of methods which have enabled the high-throughput and accurate determination of the intrinsic properties of cells and have extended into a series of applications. The microfluidic method, which is based on custom-designed micro structures, is widely used to obtain the dielectric parameters [16,17,18] and mechanical properties [19,20,21,22] of cells. This method is high-throughput, easy-to-use, and rapid. However, the performance of this method is dependent on cell sizes, which may restrict wide applications of this method. Using the atomic force microscope (AFM), the mechanical properties of cells can be detected at nanoscale and used in bio-related applications [23,24,25,26,27,28]. This method holds a nano-scaled and single-cell resolution. Nevertheless, this method typically presents a lower manipulation efficiency and features direct contact with cells. Optical tweezers, utilizing an optical force that results in cell deformation, offer a way to obtain the mechanical parameters of cells and assess drug effects [29,30,31,32,33,34]. The advantages of this method mainly involve it being contact-free and single-cell resolution. However, the high-throughput detection of a number of cells is generally difficult for this method. In addition, combining several methods into one hybrid approach is another trend in research into the intrinsic properties of cells. For instance, the combination of bulk acoustic waves with microfluidic chips is a method that examines cellular motions under the acoustic wave field to enable the non-invasive and contact-free extraction of cell density and compressibility [35,36]. However, this hybrid method can only obtain the mechanical properties of cells. A method that incorporates microfluidics into a patch clamp has been designed to acquire the cell membrane capacitance at a single-cell resolution [37,38,39,40,41]. This method also holds a lower manipulation efficiency.

The integration of microfluidics with an alternating current (AC) electric field has become a popular method for extracting a variety of intrinsic properties of cells simultaneously. In general, with metal-based electrodes that give rise to a non-uniform electric field, two main AC electrokinetic-based mechanisms are produced: electro-rotation (ROT) and dielectrophoresis (DEP). DEP corresponds to a non-uniform and non-rotational electric field and ROT to a rotational electric field. With the merits of being label-free, contact-free, and non-invasive, DEP and ROT make for a more versatile alternative to extract the intrinsic parameters of cells than other competing lab-on-a-chip techniques. A series of extracted mechanical properties of cells, such as deformation, viscoelasticity, and stiffness, are reported [42,43,44,45,46] by these two methods. Most importantly, DEP and ROT have a good prospect for use in the extraction of such dielectric properties of cells as membrane capacitance/conductance and cytoplasm conductivity/permittivity and, thereby, in bio-related applications including the identification of target cells and the detection of cell states [47,48,49,50,51,52,53]. Optically-induced dielectrophoreis (ODEP) [54,55,56], another DEP-based mechanism, is also proposed to extract the density and mass [57,58] as well as the electrical [59,60,61] and mechanical properties [62,63,64,65] of cells. With a working principle similar to that of the DEP method, this method also relies on an AC non-uniform electric field to polarize and drive cells. The only difference between the two is that ODEP can make efficient use of optically-projected images to produce virtual electrodes, meaning that this method can dynamically and programmatically manipulate cells without requiring any metal electrodes.

In this paper, we review the advances in the use of an AC electrokinetic-based microfluidic platform for determination of cell dielectric properties. First, we give a detailed explanation about the ways ROT and DEP work in extracting cell dielectric properties, followed by an introduction to differently structured ROT chips. Then, we describe how the dielectric properties of cells are determined and their applications in biomedical and bioengineering fields. Finally, we outline the current status and future challenges for the AC electrokinetic-based extraction of cell dielectric parameters.

## 2. Chip Structure and Working Principle

**Figure 1:**
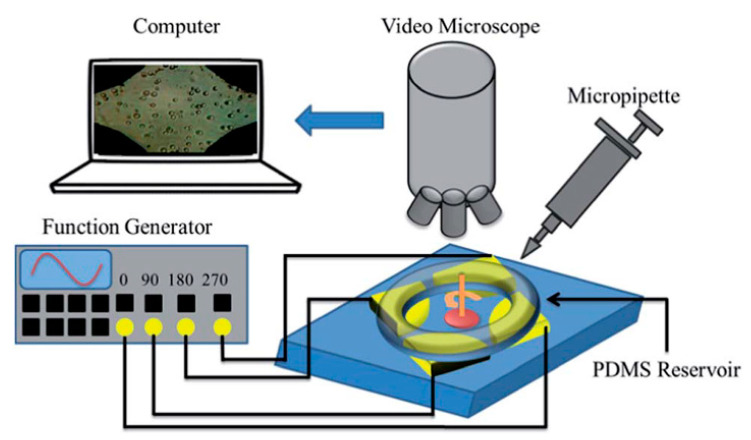
Typical schematic illustration of an electro-rotation (ROT) chip. Reproduced with permission from Bahrieh et al., RSC Adv. **4**, 44879 (2014). Copyright 2014 RSC Publishing.

Figure 1 is a typical schematic illustration of a ROT chip [66]. Four metal-based electrodes orthogonal to each other were fabricated by conventional micro-lithographic techniques and formed a crisscross pattern. The four electrodes were each wired to an AC bias potential with a phase difference of π/2. In this way, a rotating non-uniform electric field would be produced, which generated a torque exerted onto suspended cells and resulted in the ROT phenomenon. It is worth noting that if there was no voltage with phase difference among the quadrupole electrodes, no ROT would be generated, which was reported previously by our group [67]. The time-average torque is expressed as [68]:

(1) 〈ΓROT〉=−4πεmr3Im[fCM]|E→rms|2fCM=εc*−εm*εc*+2εm*,

where Im[*f_CM_*] is the imaginary part of the Clausius–Mossotti (CM) factor, *ε_m_* is the permittivity of the liquid solution, *r* is the radius of the cells, and *E_rms_* is the root-mean-square magnitude of the electric field. ε^*^ is the complex permittivity, which is expressed as ε^*^ = *ε* – j*σ*/(2π*f*), where *f* is the frequency of the externally applied AC bias potential and *σ* is the conductivity. The subscript *c* denotes cells. Based on a single-shell polarization model for a cell, *ε*_c_^*^ is further defined as[69]:

(2) $$\varepsilon_{c}^{*}=\varepsilon_{mem}^{*}\frac{{\left(\frac{r}{r-d}\right)}^{3}+2\frac{\varepsilon_{cyto}^{*}-\varepsilon_{mem}^{*}}{\varepsilon_{cyto}^{*}+\varepsilon_{mem}^{*}}}{{\left(\frac{r}{r-d}\right)}^{3}-\frac{\varepsilon_{cyto}^{*}-\varepsilon_{mem}^{*}}{\varepsilon_{cyto}^{*}+\varepsilon_{mem}^{*}}},$$

where *d* is the thickness of the cell membrane, and the subscripts *mem* and *cyto* denote cellular membrane and cytoplasm, respectively. In this case, Im[*f_CM_*] is further rewritten as [69]:

(3) Im[fCM]=2πfτmw[(εc−εmεc+2εm)−(σc−σmσc+2σm)]1+(2πfτmw)2τmw=εc+2εmσc+2σm.

In addition, a more complex double-shell model is proposed to elucidate the polarization of cells using Equation (2) for each shell [70]. In the double-shell model, the inner layer is the nucleus with the associated radii *r*_n_-*d_n_*. The complex permittivities of the nuclear membrane and nucleoplasm are denoted as ε_nb_^*^ and ε_np_^*^, respectively. The nuclear layer is separated from the cytoplasm layer by a nuclear membrane with a thickness of *d_n_*. Accordingly, ε_c_^*^ is defined as:

(4) $$\varepsilon_{c}^{*}=\varepsilon_{mem}^{*}\frac{2\left(1-\nu_{1}\right)+\left(1+2\nu_{1}\right)E_{1}}{\left(2+\nu_{1}\right)+\left(1-\nu_{1}\right)E_{1}},$$

where *ν*_1_ = (1-*d*/*r*)^3^ and *E*_1_ is expressed as:

(5) $$E_{1}=\frac{\varepsilon_{cyto}^{*}}{\varepsilon_{mem}^{*}}\frac{2\left(1-\nu_{2}\right)+\left(1+2\nu_{2}\right)E_{2}}{\left(2+\nu_{2}\right)+\left(1-\nu_{2}\right)E_{2}},$$

where *ν*_2_ = (*r_n_*/(*r*-*d*))^3^ and *E_2_* is expressed as

(6) $$E_{2}=\frac{\varepsilon_{np}^{*}}{\varepsilon_{nb}^{*}}\frac{2\left(1-\nu_{3}\right)+\left(1+2\nu_{3}\right)E_{3}}{\left(2+\nu_{3}\right)+\left(1-\nu_{3}\right)E_{3}},$$

where *ν*_3_ = (1-*d_n_*/*r_n_*)^3^ and *E_3_* = ε_np_^*^/ε_nb_^*^.

As shown in Equation (1), the torque is proportional to the imaginary part of the CM factor related to the intrinsic properties of the cells. Hence, the dielectric parameters of the cells can be extracted by the best fitting of the minimization of the root-mean-square error between the experimentally measured ROT spectra of the cells of interest and Equation (3). The ROT measurements were generally performed by manually timing the rotational speeds of cells using a stopwatch [71,72,73]. Additionally, a series of computer-based machine vision algorithms were reported to accurately and automatically determine the rotational speeds of cells [65,74,75]. By using a frame-to-frame dynamic tracking method, the measurement of a single cell’s in-plane rotation was automatically achieved [74]. The automatic acquisition of the rotational speeds of cells involving the in-plane and out-of-plane was also reported by optimizing the sum of the absolute difference between a reference frame and the current one [75]. Our group also realized the accurate extraction of the rotational speed of cells using an image-matching algorithm automatically determining the reference frame [65].

**Figure 2:**
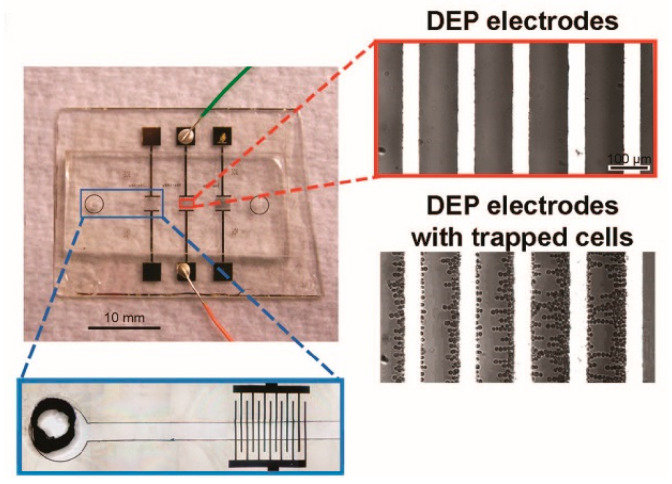
Picture of a real dielectrophoresis (DEP) chip and experimentally captured images of DEP electrodes and trapped cells using DEP. Reproduced with permission from Flanagan et al., Stem Cells **26**, 656 (2008). Copyright 2008 Wiley Publishing.

In general, an array of metal-based electrodes powered by an externally applied AC bias potential with no phase difference would produce a non-uniform electric field resulting in cell polarization, as shown in Figure 2 [76]. The interaction between the cell and the non-uniform electric field would generate a DEP force exerted onto the cell, which is expressed as [77]:

(7) 〈F→DEP〉=2πr3εmRe[fCM]∇|E→rms|2,

where Re[*f_CM_*] is the real part of the CM factor.

The changed AC frequency would lead to either a positive or negative DEP force acting on the cells, depending on the dielectric parameters of the cells and the liquid solution used. Specifically, when the Re[*f_CM_*] value was higher than zero under a given condition, the cells would experience a positive DEP force, being attracted into the edges of electrodes, as experimentally shown in the inset of Figure 2. Alternately, when the Re[*f_CM_*] value was lower than zero, they would experience a negative DEP force, being pushed away from the electrodes. In this case, there existed a critical frequency that led the DEP force to go from positive to negative or vice versa (i.e., Re[*f_CM_*] = 0), which was named the crossover frequency. The crossover frequency of cells could be derived as [60]:

(8) $$f_{crossover}=\frac{\sqrt{2}}{2\pi rC_{mem}}\sigma_{m}-\frac{\sqrt{2}G_{mem}^{*}}{8\pi C_{mem}},$$

where *C_mem_* and *G_mem_* denote the cell membrane capacitance and conductance, respectively. Furthermore, the crossover frequency would increase proportionally with the increase in liquid conductivity with a slope and a *f_crossover_*-axis intercept. Hence, the cell membrane capacitance and conductance could be further deduced as [60]:

(9) $$\begin{array}{l} C_{mem}=\frac{\sqrt{2}}{2\pi r\times slope}\\ G_{mem}^{*}=-\frac{4\times {\mathrm{intercept}}_{f_{crossover}}}{r\times slope} \end{array}.$$

**Figure 3:**
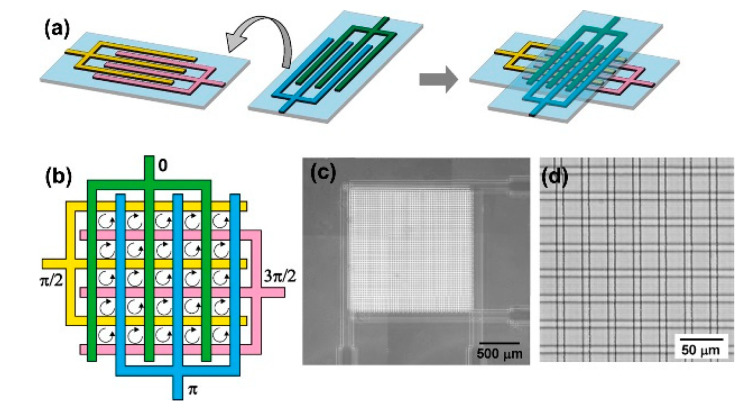
Schematic illustration of a ROT chip. (**a**) Two glass substrates coated with interdigitated array electrodes were arranged orthogonally and face-to-face; (**b**) a rotating electric field was produced by applying an electrical connection with a π/2 phase difference between adjacent electrodes; (**c**) overview of the ROT chip; (**d**) enlarged view of the ROT chip. Reproduced with permission from Ino et al., Sensors Actuators B Chem. **153**, 468 (2011). Copyright 2011 Elsevier Publishing.

In addition, a ROT chip with a millimeter-level working area was developed, as shown in Figure 3 [78]. This ROT chip was a sandwich structure formed by the top and bottom pieces of glass. Three-dimensional interdigitated array electrodes were fabricated onto the two pieces of glass and AC bias potentials with a π/2 phase difference between adjacent electrodes were also applied. A polyester film serving as a spacer was placed between the two pieces of glass. Then, a rotating electric field was produced by arranging those two interdigitated array electrodes orthogonally and face-to-face. Accordingly, a large number of measurement chambers, i.e., 2401 regions, were simultaneously created with one ROT chip. The total area of this ROT chip reached 1.74 mm^2^. This type of ROT chip made a significant improvement in its parallel and high-throughput measurement capability. 

**Figure 4:**
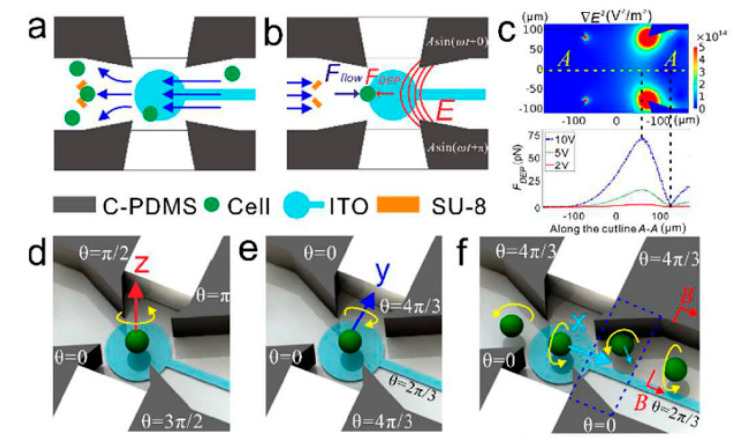
Working principle of a 3D cell ROT chip. (**a**) One single cell hydrodynamically trapped by a SU-8-based unit; (**b**) a trapped single cell was released by back flow and stayed in the rotation chamber after a negative DEP force was applied from the two electrodes; (**c**) simulation result of the negative DEP force; (**d**–**f**) are AC signals for cell rotation along the Z-axis, Y-axis, and X-axis, respectively. Reproduced with permission from Huang et al., Lab Chip **18**, 2359 (2018). Copyright 2018 RSC Publishing.

A 3D cell ROT chip capable of measuring biophysical properties and reconstructing the 3D cell morphology was proposed [79]. The working principle of the chip, including single-cell loading and 3D rotation, is shown in Figure 4. Generally, this chip was composed of four vertical carbon-black-nanoparticle-PDMS (C-PDMS) electrodes for connecting one end of the AC bias potential, one bottom indium tin oxides (ITO) electrode for connecting the other end of the AC bias potential, and one V-shaped constriction fabricated by SU-8 for a single cell trap/release. The gap of the custom-designed SU-8-based trap module was 10 μm, meaning that only one single cell could be trapped between the two pillars. Accordingly, when the applied fluid flow drove the cells in one way, as shown in Figure 4a, only the cell with a size matching the gap would be captured in the trap unit. After that, a back flow was applied to move the trapped cell away from the V-shaped constriction to the rotation chamber. Simultaneously, a negative DEP force was produced against the fluid drag force to stabilize the cell, as shown in Figure 4b. Figure 4c shows the simulation result of the negative DEP force, serving as a guide for how the negative DEP force could balance the drag force to successfully fix one single cell into the rotation chamber. Once one single cell was located in the rotation chamber, 3D rotation could be realized along three axes (Figure 4d–f) independently by switching proper AC signals on. As shown in Figure 4d, four sidewall electrodes were applied with four AC signals of equal amplitude but a phase shift of π/2, respectively, and the ITO electrode was set as floating. Then, the cell would experience in-plane rotation along the Z-axis. When five AC signals of equal amplitude but a selected phase shift were applied to the five electrodes, the cell would experience out-of-plane rotation along the Y-axis (Figure 4e) and X-axis (Figure 4f). The in-plane cell rotation spectrum was employed to determine the dielectric parameters, and the out-of-plane cell rotation was demonstrated to be able to reconstruct the 3D cell geometrical morphology. In this study, the microscope dynamically captured a series of cell rotation images in two dimensions: X-axis and Y-axis. Hence, the cell rotation was in-plane when it occurred along the Z-axis. However, when the rotation occurred along both the Y-axis and the X-axis, it was out-of-plane in terms of the field of view of the microscope. Accordingly, with out-of-plane rotation that allowed capturing the 3D cell motion, a stack of cellular contour images was observed in several rounds by keeping the cell rotation at the lowest possible speed and using the maximum frame rate of the camera. Using these images, the 3D cell geometrical morphology was reconstructed. As this study showed, the cells would not simultaneously experience ROT and DEP under the same condition that involved custom-designed electrode structures and an externally applied AC bias potential. This is because a rotational electric field in which four electrodes are orthogonal to each other with a π/2 phase-shift between any adjacent electrodes is required to generate ROT onto the cells. Under this condition, no DEP would be produced onto the cells, in that the π phase difference between a pair of electrodes or adjacent electrodes is essential to produce DEP.

**Figure 5:**
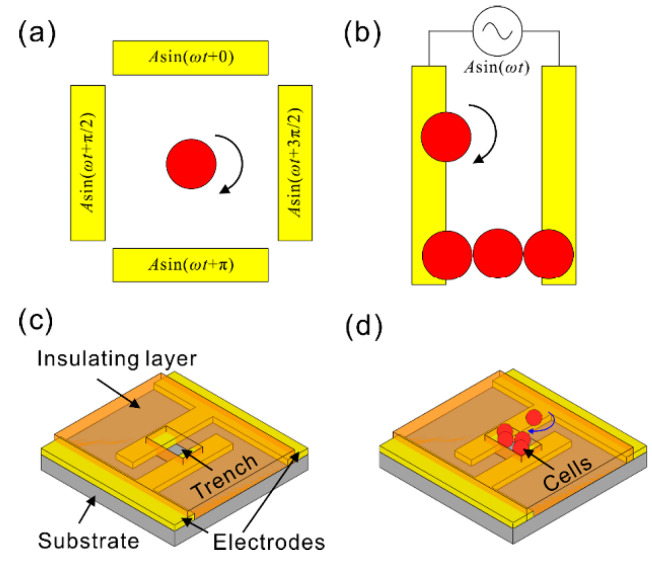
(**a**) A conventional four-electrode ROT chip; (**b**) the proposed ROT chip consisting of two electrodes and induced cell electrodes; (**c**) 3D structure of the proposed ROT chip; (**d**) operation mode of the cell electrodes and the trapped cells rotating on an insulating layer. Reproduced with permission from Huang et al., Electrophoresis **40**, 784 (2019). Copyright 2019 Wiley Publishing.

A two-electrode-based ROT chip capable of rotating cells in a microfluidic platform was proposed, as shown in Figure 5 [80]. 

Unlike conventional ROT chips that use four electrodes to produce a rotating electric field in Figure 5a, this design, as shown in Figure 5b, only featured two planar and parallel electrodes, with trapped cells used as extra electrodes. This ROT chip consisted of two electrodes, a trench-patterned insulating layer, and microfluidic channels, as shown in Figure 5c. The trench-patterned insulating layer was fabricated on the substrate. Firstly, a positive DEP force was generated by the two parallel electrodes, which moved and trapped cells into the mechanical trenches, as shown in Figure 5d. At this point, the cells polarized due to a non-uniform electric field could in turn affect the distribution of the electric field; the trapped cells in the trenches came into direct contact with the two planar electrodes, which resulted in a phase delay due to their capacitance. Based on this theory, the trapped cells in the trenches could be formed into a line which behaved as an isolated electrode and interacted with the two parallel electrodes, thereby producing a rotating electric field. Consequently, cells near and outside of the trenches could be rotated, and the rotation speed was demonstrated to be adjustable by tuning the electric signal. They simulated the influence of various factors on the ROT model to evaluate the AC signal parameter range, involving the AC frequency and amplitude. Additionally, the influence of the trench-patterned insulating layer thickness on the electric field was analyzed to fix the electrode structure. They further confirmed that this design of this ROT chip could produce a rotational electric field driving the cell to rotate through simulation. Using Equations (1)–(3), the ROT chip proposed in this study realized the extraction of the dielectric properties of cells, eliminating the four-electrode requirement while achieving the same functionality.

## 3. ROT-Based Extraction of Cell Dielectric Parameters and Its Applications

Table 1 shows a summary on the extraction of cell dielectric parameters using ROT.

**Figure 6:**
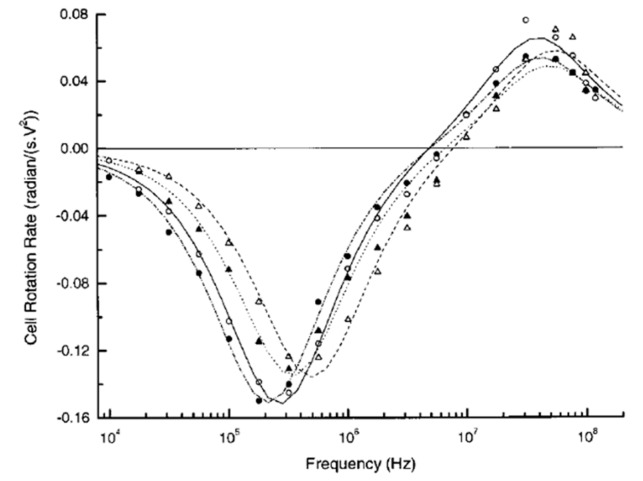
ROT spectra of B-lymphocytes (▲), T-lymphocytes (Δ), monocytes (○), and granulocytes (●) in an isotonic solution. Reproduced with permission from Yang et al., Biophys. J. **76**, 3307 (1999). Copyright 1999 Elsevier Publishing.

Through ROT experiments and Equation (3), the dielectric parameters of the four main leukocyte subpopulations, including B- and T-lymphocytes, monocytes, and granulocytes, were extracted [71]. The ROT spectra of the cells were normalized against the square of an externally applied AC bias potential. The results showed that the cells rotated clockwise when the frequency was below 6 MHz and anticlockwise when it was above 6 MHz, as illustrated in Figure 6. From Equation (1), the cell would present a clockwise rotation if the torque in Equation (1) was higher than zero; instead, an anticlockwise rotation of the cell would be observed when this torque was lower than zero.

Furthermore, the clockwise peaks were generally located around 350 kHz for B- and T-lymphocytes, 200 kHz for monocytes, and 300 kHz for granulocytes. However, the anticlockwise peaks occurred at frequencies around 40 MHz for all leukocytes. Then, the dielectric parameters of the cells—i.e., the cell membrane capacitance and the cytoplasm permittivity and conductivity—were acquired using the best curve-fitting method. The biggest membrane capacitance was found in monocytes, and the smallest one in T-lymphocytes. In addition, B-lymphocytes exhibited a larger membrane capacitance and cytoplasm permittivity and conductivity than the T-lymphocytes. This finding offers an alternative method to separate and purify cells of similar sizes. An example of this application is that ROT was employed to characterize the cell membrane capacitance changes in hypotonic solutions, which provided a non-invasive way to investigate the exocytosis-like mechanism [72]. Furthermore, the use of ROT spectra for detecting cell membrane capacitance changes, thereby enabling the real-time monitoring of cell apoptosis [87] and virus-infected cells [88] and the assessment of multidrug-resistant human leukemia cells [66], was also reported.

**Figure 7:**
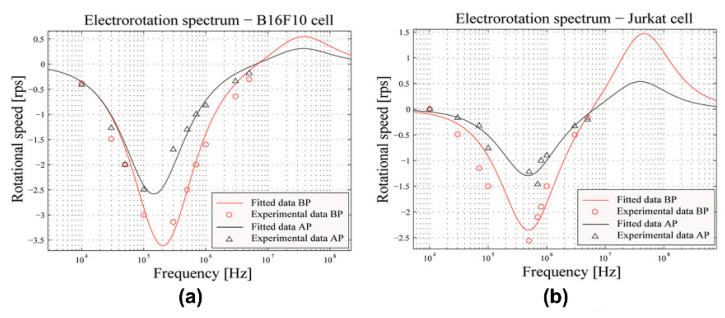
ROT spectra of the B16F10 (**a**) and Jurkat (**b**) cell lines before and after the application of a pulsed electric field. Reproduced with permission from Trainito et al., Electrophoresis **36**, 1115 (2015). Copyright 2015 Wiley Publishing.

Cell membrane permeabilization permits the transfer of extracellular molecules into the cytoplasm. A method that combines a pulsed electric field and ROT for the in situ characterization of the permeabilization of a single cell was reported [90]. Typically, the application of a pulsed electric field would lead to the permeabilization of cells; then, the cells before and after the pulse (BP and AP) application could be monitored and identified by investigating their dielectric parameters using ROT spectra. Here is an explanation of how the method worked in detail. An AC bias potential was firstly applied to generate a DEP force to trap a single cell and move it to the central area of a four-electrode pattern. Then, a rotating electric field was switched on to generate a rotating electric field that induced ROT in this single cell. The dielectric parameters of the cells were extracted using the ROT spectra and Equation (3). Once a pulsed electric field was triggered, the corresponding dielectric parameters could be acquired. In this method, the Jurkat and B16F10 cell lines were selected as the target cells; their ROT spectra are shown in Figure 7. After the application of a pulsed electric field, a decrease in the rotational speed of these two types of cells could be observed all across the ROT spectra. This should be attributed to the changes in the cell dielectric parameters caused by the cell permeabilization, which verified ROT’s ability to monitor the level of cell permeabilization. In addition, how much this method helped with improving the drug delivery efficiency could be detected using ROT.

**Figure 8:**
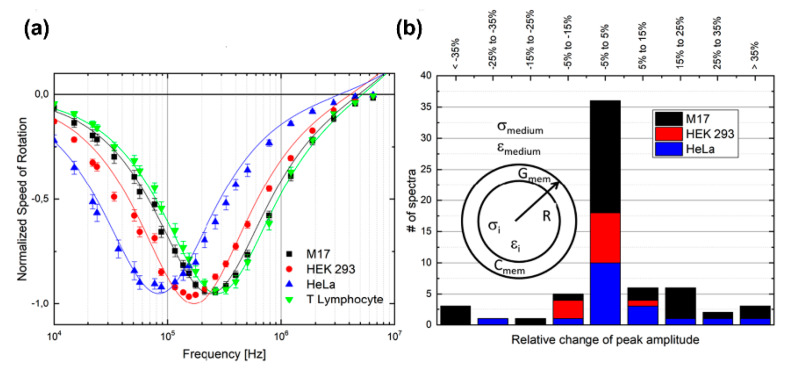
(**a**) ROT spectra of four cell types; (**b**) evaluation of the differences between peak frequencies before and after 5 minutes of exposure to the rotating electric field. Reproduced with permission from Keim et al., Electrophoresis **40**, 1830 (2019). Copyright 2019 Wiley Publishing.

The simultaneous ROT determination of the dielectric parameters of multiple individual cells was reported [93]. Multiple individual cells were trapped in 39 separate and arrayed micro cages that could selectively release the cells by adjusting the AC power parameters. The suspended cells were injected into the chip by a fluid flow. Initially, two signals with an amplitude of 1 V and a phase shift of π and another two signals with an amplitude of 5 V and a phase shift of π were applied to the entrance and exit of the ROT chip, respectively. Hence, a lower DEP barrier was created at the entrance and a higher DEP barrier at the exit. This allowed the cells to pass through the entrance barrier, but they were trapped in the rotation chamber due to the limit of the higher exit barrier. After a cell was trapped in a single-cell micro cage, the voltage at the entrance was increased to 5 V, blocking other cells from moving into this cage. Then, the phase shift between the adjacent electrodes was tuned to π/2 to produce ROT. After the ROT spectra were acquired, the trapped cells could be selectively released by switching off the corresponding AC signals at the exit electrodes. Figure 8a shows the ROT spectra of the four types of cells. The dielectric parameters of M17 neuroblastoma cells were reported for the first-time using ROT. Furthermore, an acquisition of the spectra of cells was performed consecutively to validate the stability of this method over time. The ROT of 63 cells was conducted before and after 5 minutes of exposure to the rotating electric field; a comparison of the peak frequency results is shown in Figure 8b. A total of 36 out of 63 cells showed a variation of less than 5%, lower than that presented by other techniques.

**Figure 9:**
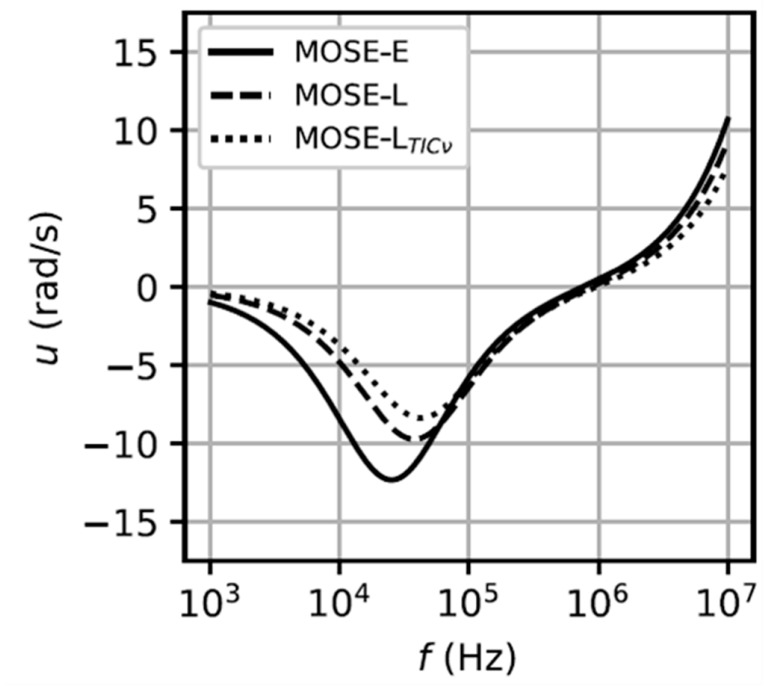
ROT spectra of the three sequentially-staged mouse ovarian surface epithelial (MOSE) cells. Reproduced with permission from Trainito et al., PLoS One **40**, e0222289 (2019). Copyright 2019 PLoS Publishing.

A method that employs ROT spectra to extract dielectric parameters for the characterization of sequentially-staged cancer cells was demonstrated [70]. The mouse ovarian surface epithelial cell line (MOSE) at three stages of malignancy—from an early stage (MOSE-E), to a malignant stage (MOSE-L, slow-developing disease), to a late and highly aggressive/invasive stage (MOSE-LTICν, fast developing disease)—was selected to analyze the corresponding ROT spectra. The results in Figure 9 indicated that the cancer cells would experience a decreased rotational speed as they became more aggressive. The cell dielectric parameters extracted from Figure 9, including the cell membrane conductance/capacitance and the cytoplasm conductivity, all increased as the phenotypic malignancy increased. This finding demonstrated the feasibility of using ROT as a label-free and non-invasive means to determine the dielectric parameters and thus characterize the cancer malignancy and progression.

## 4. DEP/ODEP-Based Determination of Cell Dielectric Parameters and Its Applications

Table 2 presents a summary of the determination of cell dielectric parameters using DEP/ODEP.

Using the crossover frequency of cells, a method that identifies dying and dead cells without using any cell-type biomarker to label cells, was proposed [104]. It was demonstrated that the dielectric properties of dying and dead yeast cells were highly dependent on the method employed to induce cellular death. In sum, methods having direct effects on cell membrane permeability would result in large changes in cell dielectric parameters; however, those having little effects on cell membranes would cause few or no changes in the cell dielectric parameters. In this study, yeast cells being exposed to two lethal environmental stresses—i.e., thermal and chemical—were investigated. The results showed that heating indirectly affected the cell membrane, which led dead cells to show similar dielectric properties to live cells; additionally, cells killed with iso-octanol at different concentrations exhibited a lower internal conductivity than those killed with heating.

**Figure 10:**
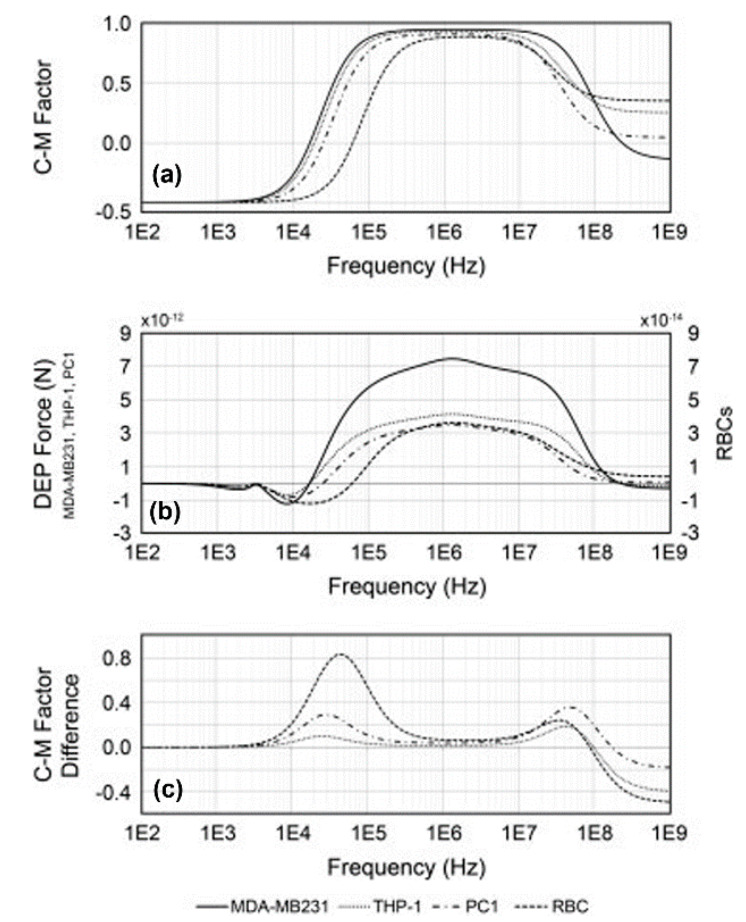
(**a**) The real part of the Clausius–Mossotti (CM) factor of MDA-MB231, THP-1, PC1, and RBCs; (**b**) DEP forces exerted on MDA-MB231, THP-1, PC1, and RBCs with respect to AC frequency; (**c**) difference in CM factor between MDA-MB231 (solid), THP-1 (dotted), PC1 (dash-dot), and red blood cells (RBCs) (broken lines). Reproduced with permission from Sano et al., Electrophoresis **32**, 3164 (2011). Copyright 2011 Wiley Publishing.

A method that examines the dielectric parameters of cells in whole blood samples (MDA-MB231, THP-1 and PC1 cells) was demonstrated [94]. By experimentally measuring cellular response under a positive/negative DEP force with the frequency sweeping of an AC bias potential, the cellular area-specific membrane capacitances of MDA-MB231, THP-1, and PC1 cells were extracted, which were 15.18 ± 1.3, 17.19 ± 2.0, and 12.75 ± 1.8 mF/m^2^, respectively. As shown in Figure 10a, the real part of the CM factor was about the same between MDA-MB231 and THP1 cells, ranging between 100 Hz and 10 MHz; however, PC1 cells and RBCs had distinctly different CM factor curves. The corresponding DEP force exerted onto each cell is presented in Figure 10b, which shows that MDA-MB231 and THP1 cells experienced different magnitudes of DEP forces due to differences in cell membrane capacitance and radius. In addition, the PC1 cells and RBCs were smaller than the cancer cells and hence experienced a significantly lower DEP force. Figure 10c shows the difference in the real part of the CM factor between MDA-MB231, THP-1, PC1, and RBCs due to their variations in cell membrane capacitance. This method offers a feasible way to detect, enrich, and isolate rare CTCs from whole blood and could potentially facilitate patient health forecasting by distinguishing CTCs from cells in biopsy.

A DEP-based method for the characterization of the dielectric properties of microorganisms was reported [96]. The cellular cytoplasm and membrane compartments of *C. parvum*, *G. lambia*, and *C. muris* were extracted by the spectral measurement of the critical voltage for the release of trapped cells with respect to frequency. From the spectra, two peaks of critical voltage for the release of cells were obtained with respect to AC frequency, through which two DEP crossover frequencies were located for the cells. The two frequencies were regarded as the first and second critical frequencies where the real part of the CM factor became zero. Specifically, the cells exhibited opposing dielectric behaviors around the two frequencies, meaning that a positive DEP force would shift to a negative one and vice versa. Then, the dielectric parameters of the cell compartments were determined. Based on the acquired dielectric parameters, the *G. lambia* and *C. muris* samples were successfully separated at a voltage of 3 V_pp_ and a frequency of 10 MHz. Using this DEP-based capture voltage spectrum method, the dielectric properties of HT-29 colon cancer cells were obtained [95]. It was reported that the cell cytoplasm permittivity and conductivity were independent from changes in liquid conductivity; instead, the cell membrane permittivity and conductivity increased with the increase in liquid conductivity. 

**Figure 11:**
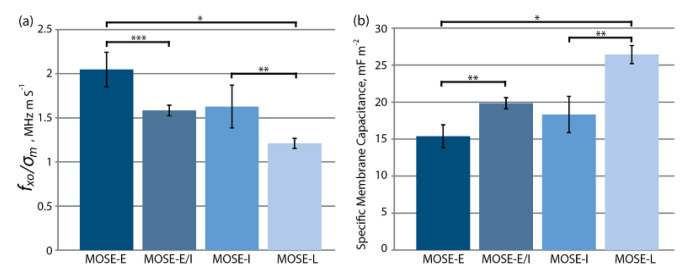
(**a**) Crossover frequencies divided by the liquid conductivity and (**b**) specific membrane capacitances measured for MOSE-E, MOSE-E/I, MOSE-I, and MOSE-L. *, **, and *** indicate *p* < 0.001, 0.01, and 0.05, respectively (n = 3). Reproduced with permission from Salmanzadeh et al., Biomicrofluidics **7**, 011809 (2013). Copyright 2013 American Institute of Physics Publishing.

A DEP-based crossover frequency method that uses the specific membrane capacitance parameter to discriminate four different stages of MOSE cells was presented [97]. Four stages of MOSE cancer cells were established by their phenotype, i.e., early (MOSE-E), early intermediate (MOSE-E/I), intermediate (MOSE-I), and late (MOSE-L). In this study, the second term of Equation (8)—i.e., the *f_crossover_*-axis intercept—could be neglected, considering the employed liquid conductivity was 0.01 S/m and the cell sizes [97]. Hence, the cellular crossover frequency was inversely proportional to the cell membrane capacitance. The cell membrane capacitance could be directly determined by measuring the corresponding crossover frequency rather than employing a series of liquid conductivities and the subsequent curve-fitting function. The measured crossover frequencies were divided by the liquid conductivity in each performed experiment to give a comparison between the four different stages of MOSE cells and the experimental runs. Figure 11 shows the crossover frequencies divided by the liquid conductivity and specific membrane capacitances measured for the four types of cells. The specific membrane capacitances extracted for MOSE-E, MOSE-E/I, MOSE-I, and MOSE-L were 15.39 ± 1.54, 19.87 ± 0.74, 18.33 ± 2.46, and 26.42 ± 1.22 mF/cm^2^, respectively (Figure 11b). The specific membrane capacitance increased from a non-malignant stage to the most aggressive stage. Further study showed that the changed cell actin and tubulin organization during the MOSE progression highly affected the cytoskeleton structures of cancer cells. In addition, using DEP-based field-flow fractionation, the correlations between the dielectric parameters and the exterior morphology of cells were presented, thereby opening up new possibilities for the application of DEP-based cell separation [99]. The apoptosis progression of Chinese hamster ovary (CHO) cells under controlled starvation was also revealed using the cytoplasm conductivity measured by the characterization of a single-cell DEP response [105]. The results indicated that apoptotic CHO cells had a cytoplasm conductivity of ~0.05 S/m, significantly lower than that of viable cells, which was ~0.45 S/m. 

**Figure 12:**
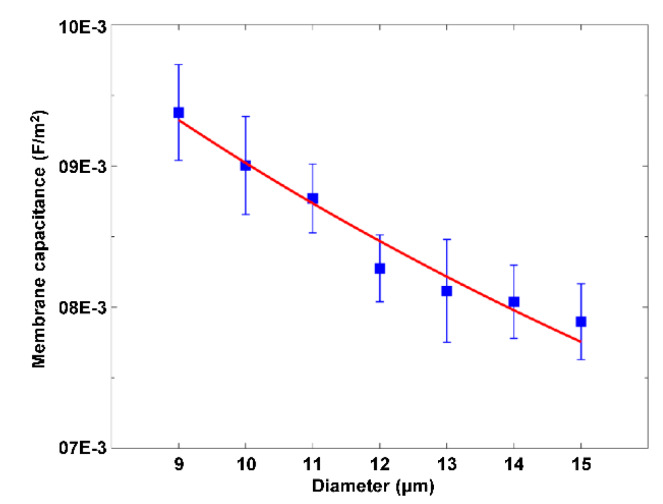
Membrane capacitance of Raji cells with respect to the diameter of these cells. Reproduced with permission from Liang et al., Biomicrofluidics **9**, 014121 (2015). Copyright 2015 American Institute of Physics Publishing.

An optically-induced DEP (ODEP)-based method proposed by our group was demonstrated to be capable of obtaining the membrane capacitance of Raji cells, which was found to vary with the diameter of these cells (Figure 12) [59].

The only difference between metal-electrode-based DEP and ODEP was how the non-uniform electric field was produced. Instead of using metal electrodes, the OEDP-based method used optically-projected patterns as virtual electrodes to trigger the photosensitive material, thereby generating a non-uniform electric field around the illumination areas in the liquid layer with suspended cells. Furthermore, our group managed to determine the membrane capacitance and conductance of the Raji cells, MCF-7 cells, HEK293 cells, and K562 cells simultaneously by using ODEP [60]. On this basis, our group also explored the application of cell membrane capacitance to the quantitative estimation of drug concentration while explaining the mechanism behind such application.

**Figure 13:**
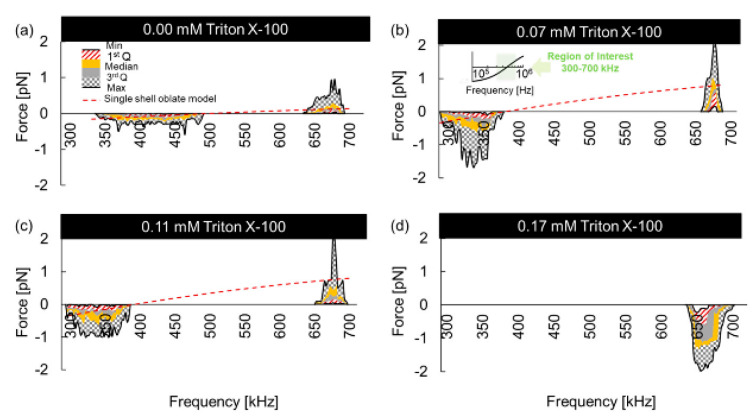
DEP profiles of (**a**) 0.00 mM (native), (**b**) 0.07 mM, (**c**) 0.11 mM, and (**d**) 0.17 mM Triton X-100-treated RBCs with respect to AC frequency (300 to 700 kHz). Reproduced with permission from Habibi et al., Biomicrofluidics **13**, 054101 (2019). Copyright 2019 American Institute of Physics Publishing.

The dielectric properties of RBCs with oblate spheroids were investigated [106]. The impacts of the Triton X-100 surfactant on human RBCs were reported in this study. The RBCs were suspended at 1.0% v/v while reaching final Triton X-100 concentrations of 0.00, 0.07, 0.11, 0.17, and 0.50 mM, respectively. Herein, the RBC suspensions in the absence of Triton X-100 (0.00 mM) were used as negative controls, and the 0.50 mM Triton X-100/RBC suspensions were employed as positive controls to achieve an expected 100% RBC lysis. The DEP responses of native RBCs and RBCs treated with low concentrations of Triton X-100 (0.07, 0. 11, and 0.17 mM) were measured experimentally to obtain the corresponding crossover frequencies. Figure 13 shows the experimental results of the DEP responses of native and Triton X-100-treated RBCs. When the Triton X-100 concentration increased, the corresponding crossover frequency decreased. No crossover frequency was observed when the concentration was 0.17 mM, which was due to the occurrence of a negative DEP force for all frequencies. Then, the membrane capacitances extracted for the 0.00 mM (native), 0.07 mM, and 0.11 mM Triton X-100-treated RBCs were 11.51, 14.05, and 13.61 mF/m^2^, respectively. One possible explanation given in the study was that the membrane permittivity of the Triton X-100-treated RBCs resulted in interfacial polarization changes. These findings revealed that low concentrations of surfactant altered the polarization of the RBCs. This study also suggested the necessity to test surfactant molecules separately in order to accurately determine cell properties and to engineer portable and reliable electrokinetic chips.

**Figure 14:**
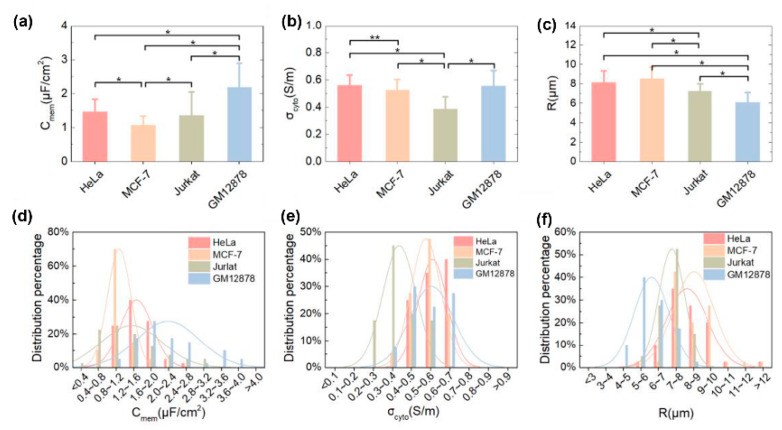
(**a**) Membrane capacitances, (**b**) cytoplasm conductivities, and (**c**) radii extracted for Hela, MCF-7, Jurkat, and GM 12878 cells. Significant difference: *p < 0.01, **p < 0.05. Distribution percentages of (**d**) membrane capacitance, (**e**) cytoplasm conductivity, and (**f**) radius for Hela, MCF-7, Jurkat, and GM 12878 cells. Reproduced with permission from Zhang et al., Sensors Actuators B Chem. **304**, 127326 (2020). Copyright 2020 Elsevier Publishing.

A DEP model reduction approach for the real-time and in situ determination of the dielectric properties of four typical cell lines—i.e., adherent cells (HeLa and MCF-7), suspension cells (Jurkat and GM12878), cancer cells (HeLa, MCF-7 and Jurkat), and normal cells (GM12878)—was presented [98]. After the redundant parameters were removed from the DEP formula, the remaining ones were decoupled to establish a solvable measurement model that allowed the direct extraction of the cell radius, membrane capacitance, and cytoplasm conductivity. In addition, the AC frequencies of DEP were optimized, which led to significantly improved measurement accuracy and efficiency. Figure 14a–c shows the determined membrane capacitances, cytoplasm conductivities, and radii, respectively. The results indicated that there was a significant difference between the normal cells and cancer cells in membrane capacitance, meaning that this parameter could be used as a biomarker to identify and isolate cancer cells from normal cells. Furthermore, the cytoplasm conductivity of Jurkat cells showed a significant difference from that of the other three types of cells. The GM12878 cells had considerably lower radii than and also showed a significant difference from the other three types of cells, meaning that the cancer cells were typically larger than the normal cells. The distribution percentages of the parameters for the four types of cells are shown in Figure 14d–f. The curve-fitting function followed a normal distribution, which clearly indicated the differences between the four types of cells.

## 5. Conclusions and Prospects

This paper aims to give a comprehensive and systematic view of the advances in the determination of cell dielectric parameters using the ROT, DEP, and ODEP methods. Although some of these advances come from other competing lab-on-a-chip technologies, the AC electrokinetic-based microfluidic platform is relatively more advantageous in that it is label-free, contact-free, non-invasive, easy to fabricate, and easy to use. Accordingly, we offer a broad and in-depth review of how this hybrid platform can be used to extract cell dielectric parameters as well as how these parameters can be used as label-free biomarkers in biomedical and drug research applications. In general, ROT is mainly used to extract cell dielectric parameters and, together with the biomarkers mentioned in this paper, to achieve bio-related applications. Compared with the ROT method, DEP/ODEP can not only determine cell dielectric parameters, but also separate different types of cells using those parameters. Hence, DEP/ODEP has been widely pursued by researchers around the world. Nevertheless, it is indeed difficult for one method to always be superior or far superior to others for all applications. Hence, the challenges facing ROT, DEP, and ODEP are discussed here.

When adopting ROT, DEP, and ODEP methods, the first challenge is the need to change the cell culture solution to an isotonic solution. The cell culture solution is generally conductive, which will short-circuit the ROT, DEP, and ODEP chips. Consequently, to ensure the chips work properly, an isotonic solution consisting of 8.5% (w/v) sucrose and 0.3% (w/v) glucose was proposed. However, this isotonic solution cannot serve as a cell culture solution and the cells can only remain active for several minutes. Although this isotonic solution has no negative effect on the application of AC electrokinetic-based microfluidics in bio-related fields, it may impede the integration of this mechanism with other fashionable micro/nano-robotic manipulation tools—such as AFM [107,108,109], scanning ion conductance microscopy [110,111,112], and acoustic tweezers [113,114,115]—to rapidly and simultaneously acquire the intrinsic properties of cells. The integration of DEP with AFM was demonstrated to be capable of manipulating and assembling nanoparticles accurately [116,117,118]. However, to the best of our knowledge, no published studies have provided convincing evidence for the feasibility of this integration. The incorporation of surface acoustic tweezers into DEP was presented for trapping cells and measuring cell aggregation [119,120]. However, it has not been reported whether this hybrid mechanism is capable of testing cell dielectric parameters without changing the liquid solution. In addition, although the combination of ODEP with acoustic tweezers was reported to perform cell lysis and manipulation [121,122], an isotonic solution is still required for the ODEP part, and the improvement in manipulation performance is not significant. By fabricating a pixelated phototransistor array in a photosensitive material, a phototransistor-based ODEP chip was for the first time reported to directly manipulate cells in a cell culture solution [123]. However, this ODEP chip involves a complicated fabrication process, and its integration with other tools has not been reported. A continuous medium exchange of ODEP was proposed to allow the use of a cell culture solution in place of an isotonic solution outside the ODEP chamber [124]. This hinders the integration of ODEP with other tools, and the use of an isotonic solution is still required during the ODEP manipulation.

Focus should also be put on the application of the extracted dielectric parameters of cells. Currently, the dielectric parameters are mainly intended to characterize cells at different stages in order to support drug research activities [59,60,70,90,97,125] and facilitate the separation of cancer cells from cell lines or CTCs from clinical samples by DEP [126,127,128] and ODEP [129,130,131]. These functions are still at the lab research level, and they have a long way to go to realize real-world applications and bring tangible benefits to the end user. Hence, it is critical to explore new solutions to expand the application of the extracted dielectric parameters to other bio-related fields. One solution is to separate target viruses from clinical samples by using ROT, DEP, and ODEP. Another solution is to use the various crossover frequencies to rapidly and accurately identify rare CTCs from billions of normal blood cells while ensuring no CTCs are driven out of the chip. More recently, CELLSEARCH (Menarini) Inc., Precision For Medicine LLC. (formerly known as ApoCell using ApoStream^TM^ CTC enrichment technology), and ClearCell of Biolidics Limited (formerly known as Clearbridge BioMedics) have commercialized their DEP-based technologies for the separation of CTCs. It is worth noting that Berkeley Lights Inc. has also been successful in commercializing its ODEP-based technology for the separation of cells. These commercialization successes suggest a promising prospect for the use of extracted dielectric parameters to shift from lab-level research to commercial applications.

To sum up, most new technologies will become outdated and even obsolete if they fail to be commercialized and made accessible to the end user. Thanks to numerous efforts by researchers, the AC electrokinetic-based microfluidic platform has come a long way in the biomedical and drug research fields over the past few decades. If the same efforts are to continue to be applied towards addressing the above-mentioned challenges, this mechanism will be sure to move to real-world applications.

## Figures and Tables

**Table 1:** ROT (electro-rotation)-based method for the extraction of cell dielectric parameters.

Cell Type	Dielectric Parameters					Reference
	*C_mem_* (mF/m^2^)	*σ_mem_* (μS/m)	*ε_mem_/ε_0_*	*σ_cyto_* (S/m)	*ε_cyto_/ε_0_*	
T-lymphocytes B-lymphocytes Monocytes Granulocytes	10.5 ± 3.1 12.6 ± 3.5 15.3 ± 4.3 11.0 ± 3.2	-	-	0.65 ± 0.15 0.73 ± 0.18 0.56 ± 0.10 0.60 ± 0.13	103.9 ± 24.5 154.4 ± 39.9 126.8 ± 35.2 150.9 ± 39.3	[71]
HL-60	15.4 ± 0.8	-	-	-	-	[73]
HeLa C3H10 B lymphocyte HepaRG	13.11 ± 0.11 14.73 ± 0.14 10.14 ± 0.08 15.83 ± 0.12	-	-	0.36 ± 0.05 0.31 ± 0.04 0.55 ± 0.07 0.26 ± 0.05	-	[79]
MDA231 T lymphocytes Erythrocytes	26 ± 4.2 11 ± 1.1 9 + 0.80	-	-	0.62 ± 0.073 0.76 ± 0.058 0.52 ± 0.051	52 ± 7.3 64 ± 5.9 57 ± 5.4	[81]
Friend murine erythroleukaemia cells	18.5	-	-	0.77	92	[82]
Neurospora Myeloma	4.0 4.5	-	-	> 1.0 0.068	-	[83]
MCF/neo MCF/HER2-11 MCF/HER2-18	20.9 17.0 25.7	-	-	-	-	[84]
Trophoblast Cytotrophoblast	17.8 ± 9.6 26.6 ± 6.2	-	-	-	-	[85]
Daudi NCI-H929	9.0 ± 0.4 4.2 ± 0.3	-	-	-	-	[86]
Jurkat cells	11.5 ± 1.6	-	-	-	-	[87]
Murine fibroblasts	8 ± 1	-	-	-	-	[88]
T lymphocyte B lymphocyte Granulocyte Monocyte SkBr3 A549	7.01 ± 0.91 10.33 ± 1.6 9.14 ± 1.06 11.77 ± 2.12 14.83 ± 1.74 16.95 ± 2.93	-	-	0.53 ± 0.1 0.41 ± 0.1 0.31 ± 0.06 0.37 ± 0.15 0.34 ± 0.06 0.23 ± 0.05	100 100 100 100 100 100	[89]
K562	8.93 ± 1.43	-	10.09 ± 1.61	0.32 ± 0.08	-	[66]
HEK293	7.94 ± 0.4	-	-	0.408 ± 0.019	85 ± 4	[52]
Jurkat E 6.1 B16F10	-	40 4.9	6.30 8.20	0.19 0.10	40.00 44.70	[90]
Insulin secreting cells	-	0.7043	11.3	1.3	170.3	[91]
White blood cells U937 PANC1 BxPC3	9.8 14 20.2 22.5	-	-	0.721 0.750 0.476 0.453	111 106 90 91	[92]
T-lymphocyte HEK293 Hela M17	8.05 ± 0.47 9.81 ± 0.39 17.51 ± 0.75 7.49 ± 0.39	-	-	0.5 0.5 0.84 0.5	78 78 60 78	[93]
MOSE-E MOSE-L MOSE-LTIC*ν*	-	32 52 61	30 30 30	0.90 1.0 1.3	45 45 45	[70]

**Table 2:** DEP/ODEP (optically-induced dielectrophoreis)-based method for the extraction of cell dielectric parameters.

Cell Type	Dielectric Parameters					Reference
	*C_mem_* (mF/m^2^)	*σ_mem_* (μS/m)	*ε_mem_/ε_0_*	*σ_cyto_* (S/m)	*ε_cyto_/ε_0_*	
MDA-MB231 THP-1 PC1 RBC	15.18 ± 1.3 17.19 ± 2.0 12.75 ± 1.8 10.89	-	-	-	-	[94]
HT-29	-	34.82 ± 1.36	6.01 ± 0.59	0.203 ± 0.017	61.14 ± 10.19	[95]
C. parvum G. lambia C. muris	-	0.186 2.47 0.095	9.84 12.11 4.39	0.047 0.016 0.052	61.35 65.58 60.46	[96]
MOSE-E MOSE-E/I, MOSE-I MOSE-L	15.39 ± 1.54 19.87 ± 0.74 18.33 ± 2.46 26.42 ± 1.22	-	-	-	-	[97]
Raji MCF-7 HEK293 K562	11.1 ± 0.9 11.5 ± 0.8 9.0 ± 0.9 10.2 ± 0.7	-	-	-	-	[60]
HeLa MCF-7 Jurkat GM12878	14. 6 ± 3.7 10.7 ± 2.7 13.5 ± 6.9 21.9 ± 7.1	-	-	0.56 ± 0.07 0.53 ± 0.08 0.39 ± 0.09 0.56 ± 0.11	-	[98]
BT-549 HS 578T SF-268 U251	14.4 ± 5.0 18.5 ± 5.6 27.5 ± 6.3 35.8 ± 6.5	-	-	-	-	[99]
Normal erythrocytes Parasitized erythrocytes	-	< 1 70 ± 20	4.44 ± 0.45 9.03 ± 0.82	0.31 ± 0.03 0.052 ± 0.003	59 ± 6 58 ± 10	[100]
Normal erythrocytes Parasitized erythrocytes	11.8 6–9.9	-	-	-	-	[101]
K562	9.7 (8.9–10.6)	-	-	0.28 (0.27–0.32)	-	[102]
T-lymphocytes B-lymphocytes Monocytes Neutrophils Eosinophils Basophils	13.29 ± 1.82 9.91 ± 0.80 14.23 ± 0.81 9.84 ± 0.07 9.39 ± 0.41 11.2 ± 1.25	-	-	-	-	[103]
